# Supplementary material for: MetaRibo-Seq measures translation in microbiomes
Source: Nat Commun. 2020 Jun 29;11:3268. doi: 10.1038/s41467-020-17081-z (PMC7324362; doi:10.1038/s41467-020-17081-z)
Supplement: Supplementary file 10 — Supplementary Data 7 [file 41467_2020_17081_MOESM10_ESM.zip › File2/Confidence_High_Taxonomy/259744_out.krona.html]

Javascript must be enabled to view this page.

members
magnitude
magnitudeUnassigned
count
unassigned
taxon
rank

259744\_out

19

2
superkingdom
19

19
phylum
1239

19
186801
class

19
order
186802

19
family
186803

genus

SRS015579\_contig\_number\_3784
19
33042
1

33043
species

SRS012969\_contig\_number\_32144SRS014459\_contig\_number\_37378SRS015065\_contig\_number\_24010SRS016132\_contig\_number\_4176SRS022071\_contig\_number\_5146SRS024075\_contig\_number\_2022SRS045004\_contig\_number\_12677SRS049446\_contig\_number\_1180SRS065397\_contig\_number\_10533SRS098073\_contig\_number\_16855SRS149325\_contig\_number\_14241
11


SRS063040\_contig\_number\_48569SRS1041145\_contig\_number\_contig-100\_84.184157SRS146813\_contig\_number\_25864
species
1896997
3


SRS011084\_contig\_number\_37108SRS015431\_contig\_number\_32742SRS019068\_contig\_number\_15359
species
751585
3


SRS047014\_contig\_number\_21124
species
2293096
1
